# Glucose metabolic reprogramming as a driver of immunosuppression in the tumour microenvironment

**DOI:** 10.1002/ctm2.70665

**Published:** 2026-05-21

**Authors:** Yang Wang, Yijun Lu, Jian Zhou, Xinrong Yang

**Affiliations:** ^1^ Department of Hepatobiliary Surgery and Liver Transplantation Liver Cancer Institute and Key Laboratory of Carcinogenesis and Cancer Invasion (Ministry of Education) Zhongshan Hospital Fudan University Shanghai China

**Keywords:** glucose metabolic reprogramming, immunometabolism, immunosuppression, immunotherapy, tumour microenvironment, Warburg effect

## Abstract

Immunotherapy has emerged as a transformative approach to cancer treatment, yet its clinical efficacy in most solid tumours remains limited, largely because of the immunosuppressive tumour microenvironment (TME). In this context, glucose metabolic reprogramming has emerged as a central determinant of tumour progression and immune dysfunction because it not only sustains the proliferative and biosynthetic demands of malignant cells but also profoundly reshapes immune responses within the TME. Effective antitumour immunity depends on the metabolic adaptability of effector immune cells, particularly the coordinated use of glycolysis and oxidative phosphorylation to support activation, expansion and cytotoxic function. Under the nutrient‐deprived, hypoxic and acidic conditions that characterize the TME, however, these cells undergo metabolic restriction that progressively drives dysfunction and exhaustion. By contrast, regulatory T cells, tumour‐associated macrophages and myeloid‐derived suppressor cells exhibit greater metabolic plasticity, enabling their persistence and reinforcing their immunosuppressive activity. In this review, we discuss how glucose metabolic reprogramming drives immune dysfunction through several interconnected processes, including glucose competition, lactate accumulation, reciprocal regulation between glucose metabolism and cytokine signalling, glycosylation remodelling and dynamic crosstalk with immune checkpoint signalling. Collectively, these mechanisms position glucose metabolism as a pivotal immunometabolic axis linking tumour bioenergetics to immune evasion and therapeutic resistance. A deeper understanding of this regulatory network may inform the rational development of combination strategies that integrate metabolic intervention with immunotherapy, ultimately improving therapeutic precision and the durability of clinical benefit.

**Highlight:**

1. Glucose metabolic reprogramming is a central driver of immunosuppression in the tumour microenvironment.
2. Glucose competition establishes a selective bioenergetic hierarchy that constrains antitumour immunity.
3. Lactate accumulation and reciprocal regulation with cytokine signalling amplify immunosuppressive signalling and reinforce immune exclusion.
4. Glycosylation remodelling translates altered metabolic flux into sustained changes in receptor stability, ligand recognition and checkpoint responsiveness.
5. Dynamic crosstalk with immune checkpoint signalling entrenches chronic immune dysfunction and therapeutic resistance.

## Graphical abstract

Glucose metabolic reprogramming is a central driver of immunosuppression in the tumour microenvironment, orchestrating immune dysfunction through multiple interconnected mechanisms. Glucose competition establishes a selective bioenergetic hierarchy that constrains antitumour immunity, whereas lactate accumulation and its reciprocal crosstalk with cytokine signalling further amplify immunosuppressive networks and reinforce immune exclusion. Meanwhile, glycosylation remodelling translates altered metabolic flux into sustained changes in receptor stability, ligand recognition and immune checkpoint responsiveness. Collectively, these processes are consolidated by dynamic interactions between cellular metabolism and immune checkpoint pathways, ultimately entrenching chronic immune dysfunction and therapeutic resistance.

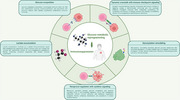

## 1 INTRODUCTION

Immunotherapy has transformed cancer treatment, but its clinical benefit remains limited to a subset of patients, particularly those with solid malignancies such as prostate and pancreatic cancers.^1^ In most cases, primary, adaptive, or acquired resistance substantially limits therapeutic efficacy and precludes durable benefit. Improving response rates and overcoming resistance, therefore, remain central challenges in contemporary cancer immunotherapy.

**FIGURE 1:**
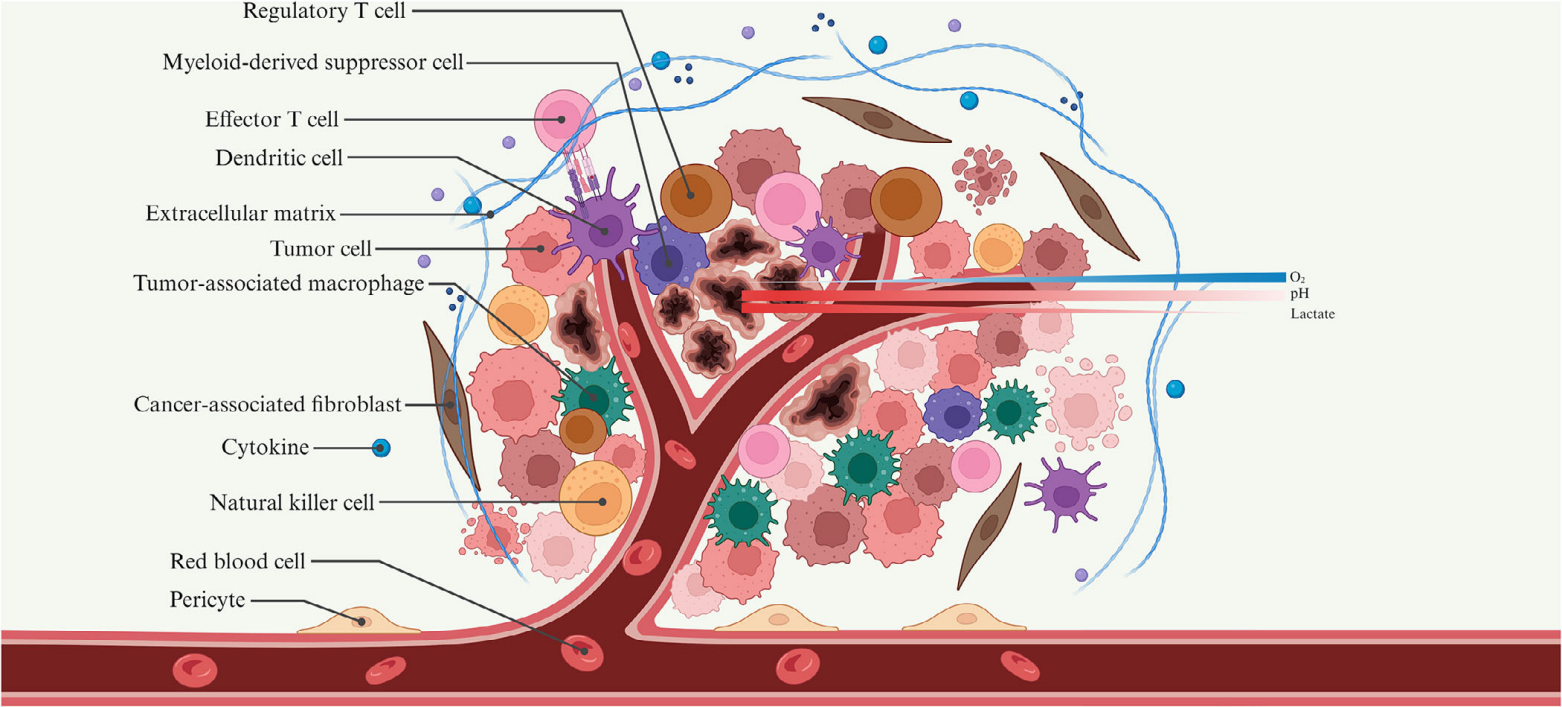
Spatial organization of immune populations and metabolic gradients in the TME. The TME comprises tumour cells and infiltrating immune populations distributed across metabolically heterogeneous regions characterized by hypoxia and extracellular acidosis.

A major reason for this limited efficacy is the profoundly immunosuppressive tumour microenvironment (TME), which is now widely recognized as one of the most persistent barriers to effective immunotherapy.^2^ The TME comprises malignant cells and a heterogeneous array of immune populations, including adaptive immune cells such as T lymphocytes and dendritic cells (DCs), as well as innate immune cells such as natural killer (NK) cells, tumour‐associated macrophages (TAMs) and myeloid‐derived suppressor cells (MDSCs) (Figure 1). Although these immune cells are continuously recruited to tumour sites in response to tumour‐derived cues, their effector functions are often compromised by metabolic, hypoxic and inflammatory stresses imposed by the local microenvironment. As a result, many immune cells become functionally exhausted, whereas others are actively reprogrammed towards tumour‐promoting phenotypes.^3^ In this context, regulatory T (Treg) cells, TAMs and MDSCs are widely regarded as the three dominant immunosuppressive cell subsets. Through coordinated mechanisms, including the secretion of inhibitory mediators, disruption of antigen presentation and suppression of effector T (Teff) cell activity, these populations collectively dampen antitumour immunity and reduce the efficacy of immunotherapy.^4^ Accordingly, a comprehensive dissection of the regulatory mechanisms by which the TME governs immune cell fate and function is essential to overcoming the current limitations of cancer immunotherapy.

As understanding of tumour immune tolerance has deepened, metabolic regulation has emerged as a critical determinant of immune cell fate within the TME.^5^ In particular, tumour cells and infiltrating immune cells engage in intense competition for glucose acquisition and utilization, thereby creating a dynamic metabolic landscape that profoundly shapes antitumour immunity.^6^ Among the metabolic programs that influence tumour immunity, glucose metabolism occupies a uniquely proximal and integrative position. It directly supports the rapid bioenergetic and biosynthetic demands of effector immune activation and serves as a central node at which diverse immunosuppressive signals converge. In this review, we discuss how glucose metabolic reprogramming in tumour and immune compartments remodels the metabolic architecture of the TME and biases immunoregulation towards suppression. By tracing the link between metabolic rewiring and immune dysfunction, we aim to provide a unifying perspective on tumour immune evasion and highlight the rationale for metabolism‐targeted combination immunotherapeutic strategies.

## 2 CELL TYPE‐SPECIFIC GLUCOSE METABOLIC REPROGRAMMING IN THE TUMOUR MICROENVIRONMENT

### 2.1 Tumour cells

Tumour cells preferentially rely on glycolysis even under normoxic conditions, a phenomenon widely referred to as the Warburg effect. By increasing glucose uptake and glycolytic flux, tumour cells secure a rapid energy supply while generating abundant metabolic intermediates for diverse biosynthetic pathways, thereby sustaining uncontrolled proliferation.^7^ This metabolic reprogramming also reduces excessive reactive oxygen species (ROS) accumulation and mitigates oxidative stress, further promoting tumour cell survival. Mechanistically, the Warburg effect is closely linked to activation of oncogenes such as KRAS, AKT and MYC, the inactivation of tumour suppressor genes including TP53, and regulation by long non‐coding RNAs (lncRNAs) and microRNAs.^8^ By contrast, hypoxia‐induced glucose metabolic reprogramming represents a broadly conserved adaptive response that is not strictly dependent on the proliferative state of cancer cells. Under hypoxic conditions, prolyl hydroxylase domain‐containing proteins (PHDs) become inactive, thereby preventing hydroxylation and subsequent proteasomal degradation of hypoxia‐inducible factor‐1 subunit α (HIF‐1α). Stabilized HIF‐1α translocates to the nucleus, heterodimerizes with HIF‐1β and activates transcription of downstream target genes.^9^ As a result, multiple glycolytic enzymes, including HK2, PFK‐1, PKM2 and LDHA, are transcriptionally upregulated, conferring substantial survival and proliferative advantages.^10^ In addition to hypoxia as an extrinsic stimulus, HIF‐1α activity is further modulated by endogenous oncogenic signalling pathways, many of which also promote aerobic glycolysis.^11^, ^12^, ^13^ Because advanced solid tumours frequently contain extensive hypoxic regions, targeting hypoxia‐driven metabolic adaptation has emerged as a promising therapeutic strategy.

Enhanced glycolytic activity leads to substantial intra‐cellular lactate accumulation, which is subsequently exported through monocarboxylate transporter 4 (MCT4), resulting in acidification of the TME.^14^ This acidic milieu selectively induces apoptosis in normal cells, promotes genomic instability and drives extracellular matrix (ECM) remodelling, collectively conferring additional survival and proliferative advantages on tumour cells.^15^

More recently, the concept of metabolic flexibility has challenged the classical Warburg hypothesis.^16^ Tumour cells can reprogram surrounding cancer‐associated fibroblasts (CAFs) to adopt enhanced aerobic glycolysis and secrete large quantities of lactate, which is subsequently imported by tumour cells through MCT1 and oxidized via oxidative phosphorylation (OXPHOS).^17^ This phenomenon, termed the reverse Warburg effect, is particularly evident in oxygen‐rich tumour regions, where it sustains an acidic extracellular milieu conducive to tumour growth, promotes angiogenesis and accelerates tumour progression. Notably, by reducing glucose consumption in well‐oxygenated regions, the reverse Warburg effect preserves glucose availability and reinforces glycolytic activity in hypoxic tumour zones. Collectively, these findings underscore the pronounced metabolic heterogeneity of tumour cells and highlight metabolic priming as a promising therapeutic strategy.^18^

Taken together, glucose metabolic reprogramming in tumour cells represents an actively orchestrated program that integrates cell‐autonomous growth with microenvironmental conditioning. Tumour cells, therefore, emerge as the primary determinants of the metabolic landscape that subsequently shapes immune behaviour within the TME.

### 2.2 Antitumour immune populations

#### 2.2.1 Effector CD8^+^ and CD4^+^ T cells

Teff cells mediate cytotoxic antitumour immunity through the secretion of IFN‐γ and TNF‐α and through the release of perforin and granzymes that induce apoptosis in tumour cells.^19^ In the quiescent state, naïve T cells primarily rely on OXPHOS to meet basal energy demands. Upon activation through TCR engagement and costimulatory signalling, they undergo a rapid metabolic shift towards aerobic glycolysis, which supports cell growth, clonal expansion and acquisition of effector functions.^20^ This metabolic reprogramming is coordinated by c‐Myc, which drives early upregulation of glucose transporter 1 (GLUT1) and HK2, and by activation of the PI3K/AKT/mTOR signalling pathway, which further enhances glucose uptake and sustains glycolytic flux.^21^, ^22^

Glycolysis plays a critical role in sustaining T cell proliferation. In gastric cancer, S100 calcium‐binding protein A8/A9 (S100A8/A9) heterodimers derived from MDSCs inhibit glycolytic activity in CD8^+^ T cells through the TLR4/AKT/mTOR signalling pathway, thereby restricting clonal expansion.^23^ Conversely, loss of the T cell‐specific protein THEMIS impairs TCR signalling, reduces nuclear translocation of nuclear factor of activated T cells (NFAT), suppresses expression of the insulin receptor and GLUT1 and ultimately results in a marked reduction in CD4^+^ T cell proliferation.^24^ Despite these advances, the precise molecular mechanisms that couple glycolytic metabolism to Teff cell expansion remain incompletely defined and represent an important area for future investigation.

Glycolysis also plays a pivotal role in shaping T cell differentiation. CD8^+^ T cells with high glycolytic activity preferentially adopt terminal effector phenotypes, whereas cells with lower glycolytic rates express memory‐associated transcription factors such as TCF‐1, LEF‐1 and BCL6, thereby promoting differentiation into long‐lived memory T (Tm) cells. In CD4^+^ T cells, metabolic modulators including empagliflozin and metformin, suppress differentiation towards proinflammatory T helper 17 (Th17) and Th1 cells by attenuating glycolytic flux and mTOR signalling, while concomitantly favouring the development of immunosuppressive Treg cells.^25^ In contrast to CD8^+^ T cells, inhibition of glycolysis does not facilitate the generation of CD4^+^ Tm cells. Instead, it enhances both the speed and magnitude of recall responses mediated by pre‐existing Tm cells.^26^, ^27^ Collectively, these findings underscore the bidirectional regulatory role of glycolysis in T cell differentiation. Under defined temporal and dose conditions, moderate suppression of glycolysis may bias T cells towards memory‐like phenotypes, thereby improving the durability of antitumour immunity.^28^

Most importantly, glycolysis is often indispensable for the antitumour activity of Teff cells. Under hypoxic conditions, HIF‐1α‐driven glycolysis is essential for sustaining IFN‐γ production, and the limited efficacy of immune checkpoint inhibitors (ICIs) has been attributed in part to insufficient HIF‐1α‐dependent metabolic remodelling in tumour‐infiltrating lymphocytes (TILs).^29^ For example, in endometrial and ovarian cancers, reduced serum levels of apolipoprotein A‐I disrupt this regulatory axis and may diminish sensitivity to ICIs.^30^ In addition, TLR7 signalling enhances glycolytic activity in CD8^+^ T cells through activation of the AKT/mTOR signalling pathway and downstream regulation of interferon regulatory factor 4 (IRF4), leading to increased expression of the transcription factor T‐bet and elevated secretion of IFN‐γ, TNF‐α and IL‐2.^31^ ATP generated by glycolysis further fuels the PI3K/AKT/forkhead box protein O1 (FOXO1) signalling pathway, thereby establishing a PI3K‐centred positive feedback loop that reinforces LDHA activity and ultimately amplifies Teff cell responses.^32^ Collectively, recent studies have identified multiple metabolic targets capable of enhancing TIL effector function, underscoring the need for further dissection of the crosstalk between metabolic and immune signalling pathways.

Interestingly, in vitro studies have shown that, at least in some model systems, expression of T cell activation markers and cytokine production are not substantially impaired under conditions of glucose deprivation.^33^ This resilience reflects the pronounced metabolic plasticity of T cells, which can compensate for reduced glycolytic flux by increasing their reliance on OXPHOS.^34^, ^35^ Given that both glycolysis and OXPHOS critically shape T cell fate and function, an important unresolved question is how these metabolic pathways are coordinated to sustain effective immunity. Recent studies indicate that OXPHOS is essential for maintaining TCF‐1^+^ stem‐like T cells during chronic antigen stimulation.^36^ Loss of OXPHOS results in downregulation of TCF‐1, upregulation of the exhaustion‐associated transcription factor TOX and accelerated progression towards terminal exhaustion. Notably, enhancing glycolysis alone is insufficient to restore stemness and self‐renewal programs. However, this metabolic adaptation may have opposing consequences in vivo. Although OXPHOS supports the maintenance of TCF‐1^+^ progenitor‐like CD8^+^ T cells, prolonged retention of an oxidative metabolic program under chronic antigen exposure and PD‐1 signalling may limit the glycolysis‐dependent effector reprogramming required for full effector differentiation and optimal cytokine production, thereby stabilizing a functionally restrained, exhaustion‐prone state.^37^, ^38^ Under hypoxic conditions in the TME, progressive mitochondrial stress may further drive these cells towards terminal exhaustion.^39^ Collectively, these findings suggest that glycolysis and OXPHOS are not simply interchangeable energy sources but complementary metabolic programs with distinct trade‐offs. Glycolysis preferentially supports acute effector function, whereas OXPHOS sustains long‐term persistence and stem‐like potential. Excessive retention of the latter program in the TME, however, may come at the expense of effector competence. Defining how to preserve this balance without allowing the TME to redirect metabolic plasticity towards dysfunction will be critical for optimizing the durability and efficacy of cancer immunotherapy.

Overall, the metabolic state of Teff cells is best understood as a demand‐coupled effector program that fuels rapid immune activation but remains highly vulnerable to persistent nutrient restriction. Accordingly, glucose metabolic reprogramming in these cells spans a continuum from active antitumour execution to adaptive compensation and, under chronic deprivation, passive functional exhaustion.

#### 2.2.2 Dendritic cells

DCs efficiently capture, process and present exogenous antigens to T cells, thereby initiating adaptive immune responses.^40^ In the resting state, DCs predominantly rely on fatty acid oxidation (FAO)‐driven OXPHOS to maintain a low‐energy homeostatic state. Upon sensing pathogen‐associated molecular patterns (PAMPs), TLR signalling rapidly activates TANK‐binding kinase 1 (TBK1), inhibitor of κB kinase subunit ε (IKKε) and AKT, promoting recruitment of HK2 to mitochondria and inducing a sharp increase in glycolytic flux.^41^ Pyruvate subsequently enters mitochondria through mitochondrial pyruvate carrier 1 (MPC1) and feeds into the TCA cycle, increasing mitochondrial membrane potential and spare respiratory capacity to support metabolic flexibility. At the same time, citrate exported from the TCA cycle into the cytoplasm via the citrate carrier (CIC) is converted to acetyl‐CoA, thereby fueling de novo fatty acid synthesis (FAS). This biosynthetic program supports expansion of the endoplasmic reticulum and Golgi apparatus, as well as production of lipid‐derived signalling mediators such as PGE2. Together, these metabolic adaptations constitute a defining feature of DC activation. Notably, emerging evidence indicates that activated DCs rely heavily on endogenous glycogen as an immediate carbon source, with glycogen catabolism supporting rapid biosynthetic remodelling during the early phase of activation.^42^

Glycolysis also plays a crucial role in DC migration. NAD^+^, a key glycolytic cofactor, facilitates filamentous actin (F‐actin) polarization and polymerization, which are essential for cytoskeletal organization and directional motility.^43^ In addition, glycolytic activation is required for oligomerization of C–C chemokine receptor type 7 (CCR7), which is indispensable for chemotaxis towards CCL19 and CCL21 and for subsequent migration to draining lymph nodes.^44^ Notably, CCR7 signalling can in turn enhance glycolytic activity in DCs, establishing a positive feedback loop that upregulates expression of glycolysis‐related genes such as LDHA.^45^ More recently, a delayed negative feedback mechanism has been identified that fine‐tunes this process.^46^ Specifically, CCR7 signalling induces expression of the lncRNA lnc‐Dpf3, which directly binds HIF‐1α during the late phase of DC migration and suppresses its transcriptional activity, thereby constraining both glycolytic flux and migratory capacity. The abundance of lnc‐Dpf3 is regulated through N6‐methyladenosine (m6A)‐mediated degradation, in which the RNA‐binding protein YTHDF2 recognizes methylated sites at the 3′ end and promotes transcript turnover under steady‐state conditions. Upon CCR7 stimulation, m6A modification is reduced, leading to stabilization of lnc‐Dpf3 and formation of a delayed metabolic feedback circuit. How this regulatory pathway operates within the TME and how it is modulated by tumour‐derived cues remain important questions for future investigation.

Glycolysis is also indispensable for the expression of surface activation markers such as CD40 and CD86 and for the secretion of proinflammatory cytokines, including IL‐12 and IL‐6, which together promote T cell proliferation and differentiation.^47^ Enolase 1 knockdown markedly impairs DC maturation and activation.^48^ Conversely, supplementation with fructose 1,6‐bisphosphate (F1,6BP) restores DC metabolic function under conditions of glycolytic inhibition, thereby supporting Teff cell responses and improving survival in tumour‐bearing mice.^49^ More recently, crosstalk between glycolytic metabolism and stimulator of interferon genes (STING) signalling has been proposed to govern the antitumour functions of DCs.^50^

At later stages of activation, bone marrow‐derived dendritic cells (BMDCs) undergo an additional metabolic transition towards a Warburg‐like phenotype. During this phase, activation of the mTORC1/HIF‐1α/inducible nitric oxide synthase (iNOS) signalling axis suppresses mitochondrial respiration. NO inhibits PHDs, thereby stabilizing HIF‐1α and further enhancing glycolytic flux to sustain bioenergetic demands.^51^ Under specific conditions, such as excessive glucose availability, sustained hyperactivation of mTORC1 induces persistent mitochondrial dysfunction, driving BMDCs into a pathologically glycolytic state that attenuates DC‐mediated T cell priming.^52^ In this context, activated T cells can compete for local glucose, thereby limiting mTORC1 signalling in BMDCs and partially relieving metabolic inhibition.^53^ Accordingly, glycolysis contributes to a negative feedback mechanism centred on glucose availability that fine‐tunes immune output during the late activation phase of BMDCs. However, these late‐stage glycolytic features cannot be readily generalized to other DC subsets that lack iNOS expression.^54^, ^55^

Overall, glucose metabolic reprogramming in DCs functions as a temporally gated licensing program for antigen presentation, migration and immune priming. Once prolonged or uncoupled from appropriate activation cues, however, this metabolic state may shift from active immunostimulation to metabolic constraint, with diminished priming capacity.

#### 2.2.3 Natural killer cells

NK cells contribute to immune surveillance through the release of cytotoxic mediators and secretion of IFN‐γ.^56^ Resting NK cells maintain a relatively low metabolic rate, whereas sustained stimulation with cytokines such as IL‐2 or IL‐12 in combination with IL‐15 induces a metabolically active state characterized by coordinated upregulation of both glycolysis and OXPHOS.^57^ Unlike the canonical mode in which OXPHOS is fuelled primarily by TCA cycle activity, activated NK cells preferentially engage the citrate‐malate shuttle (CMS).^58^ This shuttle transfers cytosolic NADH‐derived electrons into the mitochondrial NADH pool, thereby enhancing oxygen consumption and maximal respiratory capacity. At the same time, CMS‐derived oxaloacetate is reduced to regenerate cytosolic NAD^+^, thereby sustaining the activity of the glycolytic enzyme GAPDH. In this context, sterol regulatory element‐binding proteins (SREBPs) exert non‐canonical metabolic functions by upregulating key components of the CMS, such as the CIC and ACLY.^59^ In parallel, c‐Myc regulates expression of multiple metabolic modules, including glucose transporters, glycolytic enzymes and factors required for mitochondrial biogenesis. mTORC1 functions as a central metabolic node that activates SREBPs and maintains stable c‐Myc expression, thereby coordinating the metabolic programs that support NK cell effector function.^60^

A metabolic configuration characterized by coordinated upregulation of glycolysis and OXPHOS is critical for NK cell effector function. In coculture with colorectal cancer (CRC) cells, tumour cells downregulate c‐Myc expression in NK cells through competition for polyamines, thereby weakening metabolic support and reducing granzyme B and IFN‐γ expression.^61^ Consistently, in IL‐15‐activated NK cells, prolonged treatment with 2‐deoxyglucose (2‐DG) markedly suppresses proliferation and granzyme B production and is accompanied by aberrant F‐actin accumulation at the immune synapse, ultimately reducing the efficiency of target cell clearance.^62^ Under comparable conditions, the cytotoxic activity of human NK cells against K562 target cells is inhibited in a dose‐dependent manner, suggesting impaired synthesis of cytotoxic mediators or an energetic deficit that compromises granule exocytosis.^63^ Notably, in the context of IL‐15 stimulation, IFN‐γ production exhibits relative resistance to glycolytic inhibition.^64^ To define these metabolic requirements more precisely, glycolysis has been selectively constrained by substituting glucose with galactose to compare effector responses elicited by IL‐2 versus IL‐12 in combination with IL‐15.^65^ Under these conditions, glycolytic limitation has minimal effects on granzyme B expression and degranulation. However, following IL‐12 plus IL‐15 stimulation, IFN‐γ production by the CD56^bright^ subset is highly dependent on glycolysis, whereas cytotoxic activity relies predominantly on OXPHOS. By contrast, activation through receptors such as CD16 and NKG2D induces concurrent increases in glycolysis and OXPHOS, yet glycolysis is required not only for IFN‐γ expression but also directly governs degranulation, cytotoxic activity and Fas ligand expression, whereas OXPHOS primarily supports IFN‐γ synthesis.^66^ Collectively, these findings indicate that NK cells exhibit activation mode‐specific metabolic dependencies. Rapid receptor‐driven cytotoxic responses preferentially depend on glycolysis, whereas cytokine‐dominated activation states rely more heavily on OXPHOS to sustain bioenergetic demands and prolonged cytokine secretion. Apparent discrepancies in the reported roles of glucose metabolism across studies likely reflect differences in metabolic intervention strategies, variation in stimulatory contexts and intrinsic heterogeneity among NK cell subsets.

In NK cells, glucose metabolic reprogramming functions as a context‐dependent execution program that aligns bioenergetic output with the mode and duration of activation. Its functional significance, therefore, lies in sustaining rapid immune surveillance, whereas prolonged tumour‐associated stress progressively erodes effector competence.

### 2.3 Protumour immune populations

#### 2.3.1 Regulatory T cells

Treg cells maintain immune homeostasis by secreting immunosuppressive cytokines such as IL‐10 and TGF‐β, thereby inhibiting Teff cell proliferation and activation.^67^ Unlike Teff cells, Treg cells exhibit tightly constrained responsiveness to the PI3K/AKT/mTOR signalling pathway. Phosphatase and tensin homolog (PTEN) acts upstream as a lipid phosphatase that dephosphorylates phosphatidylinositol 3,4,5‐trisphosphate (PIP3), thereby preventing membrane recruitment of 3‐phosphoinositide‐dependent protein kinase 1 (PDK1) and AKT. PH domain leucine‐rich repeat protein phosphatase (PHLPP) directly dephosphorylates AKT at Ser473, thereby inhibiting its full activation. Together, these mechanisms maintain Treg cells in a low‐glycolytic state.^68^, ^69^ Restriction of mTORC1 signalling reduces glycolytic flux and attenuates TGF‐β‐mediated SMAD2 and SMAD3 phosphorylation, ultimately stabilizing FOXP3 expression and even driving terminally differentiated Th1 cells towards a FOXP3^+^ Treg cell phenotype.^70^ As the lineage‐defining transcription factor of Treg cells, FOXP3 represses c‐Myc and indirectly sustains FOXO1 activity, jointly downregulating glycolysis‐associated enzymes including GLUT1, HK2, PFKFB3 and LDHA, thereby promoting a metabolic shift towards OXPHOS.^71^ Interestingly, OXPHOS in Treg cells relies more heavily on electron transport chain complex I than on complex IV, suggesting that their metabolic advantage may derive from complex I‐mediated maintenance of a high NAD^+^/NADH ratio, which is essential for preserving cellular homeostasis.^72^

Within the TME, Treg cell metabolism becomes increasingly complex and exerts a profound influence on cellular behaviour. Tumour‐derived factors and chemokines produced by innate immune cells recruit Treg cells to tumour sites. Migration‐associated signals such as CD28 and LFA‐1 selectively activate the PI3K/AKT signalling pathway, induce mTORC2 activity and upregulate glucokinase expression, thereby enhancing glycolytic flux to fuel actin cytoskeleton remodelling and facilitate migration.^73^ HIF‐1α functions as a critical regulator of Treg cell metabolism. Its deficiency redirects Treg cells towards FAO‐driven OXPHOS, which enhances suppressive activity but compromises migratory capacity.^74^ Thus, under promigratory signalling conditions, elevated glycolytic activity promotes efficient chemotaxis and tissue recruitment, whereas oxidative metabolism plays a comparatively limited role. Achieving an appropriate balance between migratory efficiency and suppressive function is therefore critical for therapeutic modulation of Treg cells.

Once recruited to the TME, Treg cells undergo robust proliferation driven by tumour‐promoting cytokines such as TGF‐β and IL‐10 and rely on coordinated regulation of glycolysis, FAS and OXPHOS.^75^ OX40 signalling is closely associated with this highly proliferative state and induces a metabolic reprogramming program characterized by activation of SREBPs and peroxisome proliferator‐activated receptor γ (PPARγ), thereby promoting expression of metabolic genes including GLUT1, ACACB, SCD and PMVK.

Importantly, intact OXPHOS is a key determinant of Treg cell immunosuppressive function. YAP, a mechanosensor of ECM stiffness, upregulates leucyl‐tRNA synthetase 2 (LARS2) to preserve mitochondrial translation and maintain OXPHOS integrity. Loss of this axis results in heightened immune activation within the TME.^76^ Treg cells further reinforce their suppressive phenotype by upregulating arginase 2 (ARG2), which promotes arginine catabolism, reduces intra‐cellular arginine availability, inhibits mTORC1 activity and thereby strengthens immunosuppression.^77^ Mitochondrial integrity also constitutes a critical metabolic checkpoint governing Treg cell function. When lipid deprivation induces OXPHOS impairment and mitochondrial stress, a compensatory immune program is activated, leading to functional remodelling of Treg cell suppressive capacity.^78^ Conversely, Treg cells isolated from ovarian cancer exhibit elevated expression of glycolysis‐related molecules such as GLUT1 and PKM2, and activation of TLR8 suppresses glycolysis, thereby attenuating their immunosuppressive function.^79^ Collectively, these observations indicate that moderate enhancement of glycolysis facilitates rapid responses during early activation, whereas OXPHOS sustains long‐term suppressive capacity, consistent with the differential mTORC1 activity observed between effector and central Treg cells.^80^ However, current approaches for dynamic monitoring and precise modulation of Treg cell metabolic states remain limited, constraining the clinical translation of spatiotemporally targeted metabolic interventions.

Glucose metabolic reprogramming in Treg cells reflects a selectively adaptive program that preserves migration, expansion and suppressive stability across fluctuating nutrient conditions. This flexibility confers a competitive advantage within the TME, enabling persistent immunoregulation under conditions that destabilize conventional effector responses.

#### 2.3.2 Tumour‐associated macrophages

Macrophages are highly plastic immune cells that participate in pathogen clearance, tissue repair and the regulation of inflammation.^81^ Based on activation cues and functional properties, macrophages are classically categorized into proinflammatory, antitumour M1‐like phenotypes and immunosuppressive, protumour M2‐like phenotypes. M1‐like macrophages, typically induced by IFN‐γ and lipopolysaccharide, rely predominantly on aerobic glycolysis to support inflammatory effector functions.^82^ By contrast, M2‐like macrophages, induced by IL‐4 and IL‐13, preferentially use FAO and OXPHOS to sustain immunosuppressive activity and promote tissue remodelling. Within the TME, however, the metabolic programs of TAMs are extensively reconfigured and deviate substantially from this canonical polarization framework.^83^

During the early stages of tumour progression, before glucose restriction becomes pervasive within the TME, TAMs often adopt a glycolysis‐biased metabolic program that supports the development of immunosuppressive phenotypes. For example, TAMs isolated from human melanoma display high expression of M2‐associated markers such as CD163 and CD206, accompanied by activation of the AKT/mTOR signalling pathway and upregulation of glycolytic genes including GLUT1 and HK2.^84^ Similarly, hepatocellular carcinoma (HCC) induces glycolysis in TAMs through the Wnt2b/β‐catenin/c‐Myc signalling axis, thereby sustaining an M2‐like phenotype characterized by expression of CD163, IL‐10 and CCR2.^85^ Transient early activation of glycolysis in TAMs induces carbonic anhydrase 12 (CA12) expression through HIF‐1α and autocrine cytokine signalling, enhancing macrophage survival under acidic conditions while simultaneously promoting CCL8 secretion and metastatic dissemination.^86^ In parallel, diversion of glycolytic carbon into phosphoglycerate dehydrogenase (PHGDH)‐dependent serine biosynthesis sustains α‐ketoglutarate generation and mTORC1 activity in TAMs, thereby stabilizing an M2‐like immunosuppressive state.^87^ Notably, in multiple cancer types, glycolysis drives TAMs towards hybrid activation states that exhibit features of both M1‐like and M2‐like phenotypes.^88^, ^89^, ^90^ Although partially proinflammatory, these macrophages secrete cytokines including TNF‐α, IL‐1β, IL‐6 and IL‐8 that paradoxically promote angiogenesis, ECM remodelling and tumour cell proliferation, thereby enhancing tumour aggressiveness. TAM dependence on glycolysis is particularly pronounced in lactate‐rich tumour regions.^91^ In hypoxic niches, by contrast, TAMs suppress glycolytic metabolism through regulated development and DNA damage responses 1 (REDD1)‐mediated inhibition of mTORC1, shifting energy production towards FAO and OXPHOS.^92^ This metabolic reprogramming reduces their capacity to compete for glucose, thereby enabling endothelial cells to sustain glycolysis‐driven migration and vascular remodelling, perpetuating vascular abnormalities and facilitating tumour metastasis.

At advanced glucose‐restricted stages of tumour progression, several studies suggest that TAMs may shift towards OXPHOS to mitigate nutrient competition.^93^ In CRC, TAMs generally display a global reduction in enzymatic activity compared with macrophages from healthy tissue. Notably, this impairment is most evident in non‐inflammatory subsets, which are characterized by a marked reduction in GAPDH activity, whereas OXPHOS‐associated enzymes such as SDH and IDH3 fail to compensate for this deficit.^94^ These observations indicate that metabolic remodelling in TAMs does not represent a simple linear switch between glycolysis and OXPHOS. Rather, it reflects a more complex and context‐dependent rewiring of metabolic capacity. This pronounced metabolic plasticity makes TAMs both a challenging and highly promising therapeutic target. Effective interventions directed at TAM metabolism will therefore require a high degree of context specificity, tailored to the spatial, temporal and phenotypic states of TAMs during tumour evolution.

In TAMs, glucose metabolic reprogramming is best understood as an environmentally instructed polarization continuum rather than a fixed metabolic identity. Functionally, this rewiring converts local metabolic perturbation into durable tumour‐supportive immunoregulation and thereby stabilizes the suppressive architecture of the TME.

#### 2.3.3 Myeloid‐derived suppressor cells

MDSCs arise from immature myeloid precursors and regulate immune responses by suppressing Teff cell proliferation and activation and by impairing antigen presentation.^95^ They are broadly classified into polymorphonuclear (PMN‐MDSCs) and monocytic myeloid‐derived suppressor cells (M‐MDSCs), which act synergistically to dampen antitumour immunity. The immunosuppressive activity of PMN‐MDSCs is closely linked to activation of glycolysis and the pentose phosphate pathway (PPP).^96^ Glycolytic intermediates are diverted into the PPP to generate abundant NADPH, thereby providing reducing equivalents for NADPH oxidase 2 (NOX2)‐mediated ROS production and inducing antigen‐specific T cell dysfunction. By contrast, M‐MDSCs primarily mediate immunosuppression through NO production, leading to broad and sustained immune tolerance.^97^ Their metabolic program couples glycolysis with amino acid metabolism, allowing glycolytic flux to supply both the energy and reducing power required for immunosuppressive activity.

Glycolysis is also essential for MDSC survival. The glycolytic intermediate phosphoenolpyruvate (PEP) buffers excessive ROS production, thereby mitigating oxidative stress and preventing both early and late apoptosis, ultimately prolonging cell survival.^98^ Consistently, silencing GLUT3 in CD205^+^ PMN‐MDSCs induces apoptosis, as evidenced by marked upregulation of cleaved poly(ADP‐ribose) polymerase and cleaved caspase‐3.^99^, ^100^ This GLUT3‐mediated adaptation to low‐glucose conditions may confer a metabolic advantage on PMN‐MDSCs over M‐MDSCs within the TME, highlighting a potential therapeutic vulnerability for selectively attenuating PMN‐MDSC‐mediated immunosuppression.

The metabolic state of MDSCs also critically shapes their migratory capacity.^101^ During early tumorigenesis, PMN‐MDSC‐like populations exhibit enhanced spontaneous migration driven by coordinated activation of glycolysis and OXPHOS. These cells release ATP through pannexin 1 hemichannels. The released ATP activates the purinergic receptors P2Y1 and P2Y2, thereby establishing an autocrine signalling loop that sustains high migratory potential. Notably, this migratory mode is independent of increased F‐actin polymerization and instead relies on enhanced myosin light chain 2 (MLC2) phosphorylation and Rho‐associated protein kinase (ROCK) activity, consistent with a contractility‐driven mechanism. Importantly, this metabolically regulated migratory phenotype emerges before PMN‐MDSCs acquire overt immunosuppressive function, suggesting that it represents an early hallmark of pathological activation and contributes to premetastatic niche formation.^102^

Furthermore, glycolysis critically regulates the differentiation and expansion of MDSCs. Pharmacological inhibition of glycolysis with 2‐DG markedly impairs differentiation of bone marrow precursors into immunosuppressive M‐MDSCs.^103^ Tumour‐infiltrating M‐MDSCs exhibit substantially higher glycolytic activity than their splenic counterparts, accompanied by upregulation of immunosuppressive mediators such as ARG1, iNOS and PD‐L1, as well as an increased cellular frequency within tumour tissues.^104^ Enhanced glycolytic flux drives a functional transition of MDSCs from an antigen‐specific suppressive program mediated predominantly by ROS to an antigen‐non‐specific immunosuppressive state dependent on iNOS and ARG1. This metabolic shift further promotes differentiation of M‐MDSCs into immunosuppressive TAMs, reflecting a metabolically governed regulatory continuum that extends from functional remodelling to terminal differentiation.^105^

Interestingly, under conditions of chronic tumour‐induced stress, β2‐adrenergic receptor (β2‐AR) signalling reprograms MDSC metabolism away from glycolysis and towards FAO and OXPHOS, concomitantly enhancing PGE2 synthesis and establishing an alternative immunosuppressive mechanism.^106^

In MDSCs, glucose metabolic reprogramming constitutes a pathogenic adaptive program that integrates survival, trafficking, expansion and suppressive activity into a unified myeloid response to tumour‐associated stress. Their metabolic state, therefore, reflects actively maintained immunosuppressive competence rather than passive tolerance of the hostile TME.

## 3 MECHANISMS BY WHICH GLUCOSE METABOLIC REPROGRAMMING DRIVES IMMUNOSUPPRESSION

### 3.1 Glucose competition

#### 3.1.1 Constraints on antitumour immune populations

Accelerated tumour growth and vascular dysfunction generate spatially heterogeneous nutrient landscapes, creating glucose‐limited niches within the TME.^107^ In murine sarcoma models, excessive glucose consumption by tumour cells suppresses mTOR signalling in T cells, diminishes glycolytic capacity and impairs IFN‐γ production.^108^ Conversely, loss of glucose‐6‐phosphate isomerase (GPI) in tumour cells markedly enhances CD8^+^ T cell responses. However, this effect appears to arise primarily from tumour‐intrinsic glycolytic alterations that increase susceptibility to TNF‐α‐mediated bystander killing, rather than from direct relief of nutrient competition.^109^ Metabolic stress also substantially compromises the efficacy of immunotherapies. In glucose‐limited environments, chimeric antigen receptor (CAR) T cells exhibit impaired proliferation, survival and effector function, whereas metabolic engineering strategies such as enforced expression of GLUT1 or GLUT5 enhance metabolic adaptability and improve antitumour efficacy.^110^, ^111^, ^112^ By contrast, high‐glucose conditions can transiently benefit Teff cells. Human CD8^+^ T cells exposed to elevated glucose concentrations exhibit enhanced cytotoxicity in vitro, largely because of altered calcium signalling.^113^ In glioblastoma mouse models, short‐term consumption of glucose‐enriched beverages induces IFN‐stimulated gene expression in CD8^+^ T cells, enhances cytotoxic function and drives CD4^+^ T cells towards an NKG2D^+^ cytotoxic phenotype, thereby synergizing with ICI therapy.^114^ Nevertheless, excessive glucose intake increases mitochondrial ROS production in T cells, activates latent TGF‐β and promotes aberrant Th17 differentiation, ultimately exacerbating systemic inflammation and disrupting immune homeostasis.^115^ Collectively, these observations indicate that glucose availability exerts both beneficial and detrimental effects on T cell immunity in a dose‐ and time‐dependent manner, underscoring the need to define thresholds that distinguish protective from pathogenic responses.

In pancreatic ductal adenocarcinoma (PDAC), elevated tumour glycolysis correlates with reduced DC infiltration, suggesting that nutrient deprivation constitutes a major barrier to DC activation.^116^ FBP1, a key gluconeogenic enzyme, suppresses tumour glycolysis and, in turn, promotes DC activation and maturation, ultimately enhancing CD8+ T cell responses.^117^ Glucose deprivation induces ROS accumulation and oxidative stress in DCs, triggering activation of AMPK, which reduces ATP consumption and shifts metabolism towards FAO. Although this adaptation supports cellular survival, it further compromises glycolytic capacity and diminishes immunogenicity.^118^ Accordingly, AMPK has emerged as a potential target for metabolic intervention. Restoration of glucose availability can partially rescue DC function, as reflected by increased expression of CD83 and CD86, enhanced secretion of IL‐6 and IL‐12 and reduced IL‐10 production.^119^, ^120^ However, excessive glucose availability can trap BMDCs in a pathologically hyperglycolytic state, ultimately impairing antitumour immunity. Future studies should therefore define optimal glucose thresholds for DC function and quantitatively map the spatiotemporal dynamics of glucose competition between T cells and DCs within the TME.

Glucose deprivation also impairs NK cell activity. In lung and gastric cancers, elevated expression of FBP1 in NK cells is associated with functional impairment, and pharmacological inhibition of this enzyme during the early‐to‐intermediate stages of tumour progression has been proposed as a strategy to restore NK cell‐mediated immunity.^121^, ^122^ NK cell dysfunction under glucose‐limited conditions is closely linked to AMPK‐mediated energy‐sensing suppression, with additional modulation by glycogen synthase kinase‐3β (GSK‐3β) and diacylglycerol kinase‐ζ (DGK‐ζ).^123^, ^124^ Notably, prolonged glucose restriction profoundly suppresses NK cell proliferation and activation but exerts relatively modest effects on short‐term cytotoxicity.^125^ For example, within the glucose‐limited bone marrow microenvironment of patients with multiple myeloma, IL‐2‐activated NK cells retain substantial antitumour activity.^126^ These findings suggest that, under specific activation states, NK cells retain therapeutic potential even in nutrient‐deprived environments, highlighting adaptive strategies for overcoming metabolic stress within the TME.

#### 3.1.2 Adaptation of protumour immune populations

Compared with antitumour immune populations, protumour immune subsets exhibit greater resilience to nutrient competition within the TME. Tumour‐infiltrating Treg cells frequently express high levels of fatty acid transporters such as CD36 and FATP1.^127^ FOXP3 not only enables Treg cells to use long‐chain fatty acids as alternative energy substrates in glucose‐deprived microenvironments but also enhances resistance to lipotoxic stress, thereby preserving mitochondrial integrity and cellular viability under lipid‐rich conditions.^128^ In certain tumour contexts, mTORC1‐driven de novo FAS provides additional metabolic support for Treg cells.^129^ Under hypoxic conditions, HIF‐1α redirects glucose metabolism away from mitochondrial oxidative pathways, further reinforcing reliance on fatty acid‐driven mitochondrial metabolism. Together, these adaptations promote Treg cell persistence and suppressive function under conditions of glucose limitation.^130^

Similarly, TAMs and MDSCs display marked metabolic plasticity in response to nutrient stress, characterized by reduced glycolytic activity coupled with compensatory increases in FAO and OXPHOS, as discussed above. Notably, despite this apparent flexibility, TAMs and MDSCs often exhibit the highest per‐cell glucose uptake and overall metabolic activity within the TME, thereby consistently dominating local nutrient competition.^131^

Overall, glucose competition represents an upstream gatekeeping mechanism through which the TME imposes differential metabolic fitness across cellular populations. Its immunological consequence is the establishment of a selective energetic hierarchy that progressively undermines glucose‐dependent antitumour immunity while favouring cells with greater metabolic plasticity.

### 3.2 Lactate accumulation

#### 3.2.1 Suppression of antitumour immune populations

Aerobic glycolysis in tumour cells, together with that in immunosuppressive populations such as TAMs and MDSCs, results in substantial lactate accumulation within the TME. Elevated lactate concentrations consistently correlate with poor prognosis across multiple cancer types, largely because of the broad inhibitory effects of lactate on antitumour immune cells.^132^, ^133^ Lactate perturbs the intra‐cellular NAD^+^/NADH redox balance and impairs the enzymatic activity of GAPDH and PHGDH, thereby disrupting T cell metabolism and proliferation.^134^ Recent evidence further shows that lactate directly binds to an intra‐cellular motif of GLUT10, inhibiting glucose uptake in CD8^+^ T cells and suppressing their antitumour activity.^135^ Beyond its metabolic inhibitory effects, lactate uptake drives epigenetic reprogramming through histone lactylation, leading to upregulation of TOX expression.^136^ Elevated TOX subsequently promotes expression of inhibitory receptors, including PD‐1 and TIM‐3, while suppressing cytotoxic effector programs. Moreover, TOX remodels chromatin accessibility to establish an exhaustion‐specific epigenetic landscape, thereby stabilizing and reinforcing the exhausted state of CD8^+^ T cells.^137^ The identification of MCT11 as an exhaustion‐associated lactate transporter further underscores the role of sustained lactate import in maintaining this dysfunctional state.^138^ Notably, lactate exerts context‐dependent effects. At low concentrations, it can serve as a substrate for the TCA cycle and support Teff cell responses.^139^ Harnessing this dual role is therefore critical for optimizing immunotherapeutic strategies that target lactate metabolism. Consistent with this concept, recent work suggests that lithium carbonate redirects lactate metabolism towards mitochondrial oxidation, enabling lactate to function as an alternative energy source for CD8^+^ T cells under nutrient‐restricted conditions and thereby sustaining effector function.^140^

In DCs, lactate has long been recognized as a potent suppressor of antigen‐presenting capacity. Lactate downregulates surface expression of MHC class I, MHC class II and CD86, accelerates antigen degradation and disrupts cross‐presentation, collectively weakening antitumour immune responses.^141^, ^142^ In addition, lactate induces expression of the transcription factor EGR1, suppresses CD80 expression and shifts the TME from an inflammatory to a non‐inflammatory state.^143^ Mechanistically, lactate alters STAT3, ERK and p38 MAPK signalling, resulting in impaired IL‐12 production, reduced CCR7 expression and functional paralysis of DCs.^144^ Lactate also affects DC differentiation. Melanoma‐derived lactate activates SREBP‐2 signalling, promoting the generation of tolerogenic DCs.^145^

High lactate concentrations similarly impair NK cell immunity. Lactate interferes with NFAT signalling and markedly suppresses IFN‐γ production.^146^ Exposure to 15 mM lactate nearly abolishes IFN‐γ secretion by NK cells, whereas intra‐tumoral lactate concentrations can reach up to 40 mM.^147^ In addition, lactate induces protein lactylation, disrupts NAD^+^ metabolism, promotes mitochondrial fragmentation and drives excessive ROS accumulation, collectively compromising metabolic homeostasis and cytotoxic function.^148^ The acidic microenvironment resulting from lactate accumulation further exacerbates NK cell dysfunction by reducing perforin and granzyme secretion, impairing mitochondrial activity and inducing apoptosis.^149^, ^150^

#### 3.2.2 Reinforcement of protumour immune populations

Lactate provides substantial metabolic support for immunosuppressive immune subsets, thereby further facilitating tumour immune escape. Compared with Teff cells, Treg cells exhibit greater metabolic adaptability in high‐lactate environments.^151^ Treg cells can use lactate as an alternative carbon source to partially bypass glucose limitation, generating PEP to replenish glycolytic intermediates and sustain proliferation.^152^ In addition, lactate induces nuclear translocation of NFAT1, upregulates PD‐1 expression on Treg cells and leads to aberrant Treg cell activation during PD‐1 blockade, thereby suppressing CD8^+^ T cell responses.^153^ Lactate also reinforces Treg cell suppressive function through post‐translational modifications. Specifically, lactate induces lactylation of moesin at Lys72, which enhances its interaction with TGF‐β receptor I, promotes SMAD3 phosphorylation, activates TGF‐β signalling and stabilizes FOXP3 expression.^154^ Lactate can further potentiate Treg cell suppressive function by inducing histone H3K18 lactylation, which enhances NF‐κB p65 transcription and upregulates tumour necrosis factor receptor 2 (TNFR2) expression. This program is associated with increased inhibitory receptor expression and stronger suppression of CD8^+^ T cell responses.^155^ Notably, extracellular acidification itself further promotes the TGF‐β‐dependent induction of FOXP3^+^ Treg cells.^156^

Lactate also profoundly influences TAM polarization, promoting an M2‐like immunosuppressive phenotype characterized by increased expression of CD206 and ARG1 and upregulation of VEGF and IL‐10.^157^ Key signalling pathways implicated in lactate‐driven macrophage reprogramming include HIF‐1α, HIF‐2α and ERK/STAT3.^158^, ^159^, ^160^ Lactate also activates GPR132, amplifying polarization‐associated signalling and promoting tumour cell adhesion, migration and invasion in breast cancer models.^161^ PPARγ acts as a negative regulator of this process by repressing GPR132 transcription, and its activation effectively blocks lactate‐induced macrophage polarization.^162^ Lactate can also function as an odorant‐like signalling molecule sensed by the olfactory receptor Olfr78 expressed on TAMs. Mechanistically, Olfr78 forms a heterodimer with GPR132, enabling lactate sensing and driving M2‐like immunosuppressive polarization.^163^ However, the effects of lactate are not confined to direct polarization of TAMs. In CRC, tumour‐derived lactate stimulates TAMs to secrete high mobility group box 1 (HMGB1), which in turn activates epithelial–mesenchymal transition (EMT), ERK and Wnt signalling in tumour cells, thereby establishing a self‐reinforcing protumour feedback loop.^164^

In MDSCs, lactate likewise functions as an intra‐cellular signalling molecule that reinforces immunosuppression. Lactate prevents ubiquitin‐mediated degradation of the transcription factor c‐Jun, thereby enhancing cyclooxygenase‐2 expression and promoting PMN‐MDSC expansion and suppressive activity.^165^ In pancreatic cancer, lactate engages GPR81 to activate the mTOR/HIF‐1α/STAT3 signalling axis, driving MDSCs towards an immunosuppressive phenotype and further suppressing Teff cell responses.^166^ This mechanistic framework provides a plausible explanation for the clinical association between radiotherapy‐induced Warburg metabolism, lactate accumulation and therapeutic resistance.

**FIGURE 2:**
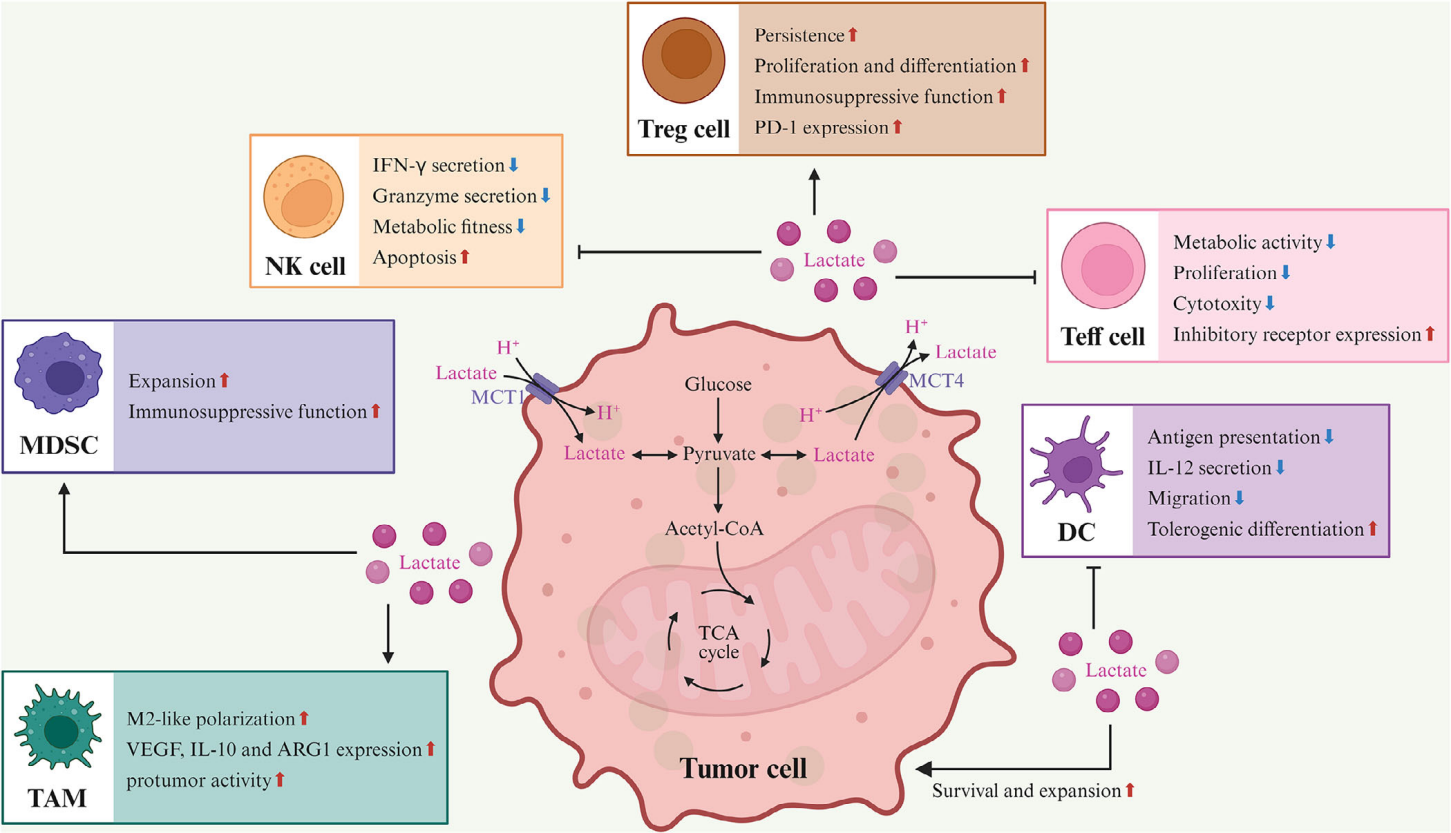
Lactate‐mediated metabolic crosstalk in the TME. Tumour‐derived lactate remodels the TME by suppressing the metabolic activity and effector functions of antitumour immune cells while sustaining the persistence and suppressive activity of immunosuppressive populations.

Taken together, lactate should be understood not merely as a byproduct of glycolysis but as a central immunometabolic signal that converts metabolic asymmetry into coordinated immunosuppression within the TME (Figure 2). By simultaneously attenuating antitumour effector programs and reinforcing protumour regulatory circuits, lactate serves as a key mediator through which altered glucose metabolism is translated into durable immune escape.

### 3.3 Reciprocal regulation between glucose metabolism and cytokine signalling

#### 3.3.1 Tumour cell crosstalk with antitumour immune populations

TGF‐β is a central mediator through which tumour cells orchestrate immunosuppressive regulation within the TME.^167^ Persistent lactate accumulation promotes activation and release of latent TGF‐β, whereas enhanced TGF‐β signalling, in turn, reinforces Warburg metabolism in tumour cells, thereby establishing a self‐sustaining positive feedback loop.^168^. HIF‐1α serves as a key regulatory hub in this process, and hypoxic conditions further potentiate TGF‐β‐driven immunosuppression.^169^ By suppressing c‐Myc‐dependent metabolic reprogramming, TGF‐β constrains early CD8^+^ T cell activation.^170^ Under sustained stimulation, TGF‐β further inhibits mTOR signalling, enforcing a relatively stable metabolic state centred on mitochondrial metabolism. Although this adaptation supports the long‐term survival of progenitor‐exhausted T cells in nutrient‐restricted environments, it does so at the expense of immediate effector function and is accompanied by progressive accumulation of inhibitory receptors, including PD‐1, TIM‐3 and LAG‐3.^171^ In addition, TGF‐β downregulates C‐X‐C chemokine receptor type 3 (CXCR3) expression on CD8^+^ T cells, thereby restricting intra‐tumoral migration.^172^ In DCs, TGF‐β‐mediated regulation of glucose metabolism preserves a tolerogenic phenotype and suppresses immunogenic maturation.^173^ TGF‐β1 also impairs DC migration to tumour‐draining lymph nodes (TDLNs), thereby facilitating lymphatic dissemination.^174^ In NK cells, TGF‐β inhibits both glycolysis and OXPHOS, resulting in diminished IFN‐γ production and reduced cytotoxic capacity, thereby further compromising antitumour immunity.^175^

IFN‐γ is a pivotal effector cytokine in antitumour immunity. Secreted by Teff cells and NK cells, IFN‐γ enhances tumour immunogenicity by upregulating MHC class I expression.^176^ However, immunosuppression within the TME markedly reduces IFN‐γ availability, with Teff cells and receptor‐activated NK cells being more profoundly affected than cytokine‐stimulated NK cells.^177^, ^178^ Neutralization of IFN‐γ signalling deprives tumour cells of the ability to induce chemokines such as CXCL9 and CXCL10, thereby limiting recruitment of CXCR3^+^ CD8^+^ T cells and NK cells.^179^, ^180^ Notably, IFN‐γ induces metabolic reprogramming in a subset of tumour cells through a NO‐dependent mechanism characterized by increased glycolytic flux and impaired mitochondrial oxidative metabolism.^181^ Distinct from canonical hypoxia‐driven Warburg metabolism, IFN‐γ‐induced NO and ROS promote HIF‐1α stabilization and activation, leading to upregulation of glycolysis‐associated genes and enhanced lactate production. Under specific conditions, elevated glycolysis and lactate accumulation further stimulate NO production, thereby establishing a feedback loop that ultimately restrains tumour cell growth. However, insufficient IFN‐γ levels within the TME preclude activation of this growth‐suppressive pathway. In parallel, IFN‐γ induces PD‐L1 expression on tumour cells, constituting a negative feedback mechanism.^182^ Attenuated IFN‐γ signalling therefore contributes to the formation of cold tumours and markedly reduces responsiveness to ICIs.^183^

IFN‐λ serves as a complementary antitumour signal to IFN‐γ and is selectively produced by conventional type 1 DCs following TLR3 activation.^184^ IFN‐λ production depends on peroxisome‐associated mitochondrial antiviral signalling (MAVS) activity and concomitant metabolic rerouting of glucose flux into the PPP. Because this process is highly sensitive to cellular nutrient availability, nutrient‐restricted conditions within the TME may impair IFN‐λ production^185^. Similar to IFN‐γ, IFN‐λ signalling positively correlates with ICI efficacy, whereas chronic suppression of this pathway accelerates tumour progression.^186^

#### 3.3.2 Tumour cell crosstalk with protumour immune populations

Tumour‐derived colony‐stimulating factor 1 (CSF1) primarily targets TAMs and promotes immunosuppression.^187^ Tumour glycolytic activity is partly linked to CSF1 secretion, an association closely related to hypoxia and lactate accumulation arising from metabolic imbalance.^188^, ^189^ Elevated CSF1 signalling supports TAM survival and suppressive function through multiple metabolic mechanisms. In the acute setting, CSF1 coordinately activates PI3K and phospholipase C signalling to regulate actin cytoskeleton dynamics, thereby promoting GLUT1 translocation to the plasma membrane and enhancing glucose uptake and glycolysis to meet immediate energetic and biosynthetic demands.^190^ Under IL‐4‐polarizing conditions, CSF1 activates the mTORC2/IRF4 signalling axis to reinforce glycolysis and sustain an M2‐like phenotype characterized by ARG1, IL‐10 and PD‐L2 expression.^191^ During prolonged differentiation, CSF1 further drives TAMs towards a metabolic state dominated by FAO and OXPHOS, thereby maintaining long‐term suppressive homeostasis.^192^ Consistently, pharmacological inhibition of the CSF1R disrupts cholesterol biosynthesis and lipid metabolic programs, underscoring the metabolic vulnerability of CSF1R signalling in TAM maintenance.^193^ Beyond metabolic regulation, CSF1 induces sialic acid‐binding Ig‐like lectin 15 (Siglec‐15) expression on TAMs, further suppressing Teff cell proliferation.^194^ In parallel, CSF1R‐mediated PI3Kγ signalling inhibits NF‐κB activation while promoting CCAAT/enhancer‐binding protein β (C/EBPβ) activity, thereby enhancing IL‐10 and ARG1 transcription and exacerbating immune evasion.^195^

KRAS‐activated tumour cells are a major source of granulocyte‐macrophage colony‐stimulating factor (GM‐CSF) and play a pivotal role in remodelling the myeloid microenvironment.^196^ In triple‐negative breast cancer (TNBC), enhanced Warburg metabolism suppresses AMPK/unc‐51‐like kinase 1 (ULK1)‐mediated autophagy, leading to stabilization of the liver‐enriched activator protein (LAP) isoform of C/EBPβ and increased secretion of GM‐CSF.^197^ GM‐CSF‐induced TAMs and MDSCs exhibit increased glucose uptake and glycolytic flux that, together with lipid metabolic pathways, establish a robust immunosuppressive phenotype.^198^, ^199^ GM‐CSF signalling additionally stabilizes antiapoptotic protein networks in myeloid cells, exemplified by increased stability of myeloid cell leukemia‐1 (MCL‐1) and upregulation of BCL‐2 family members through the PI3K/AKT and ERK signalling pathways, thereby prolonging the lifespan of otherwise short‐lived myeloid cell populations.^200^

CCL2 is another critical tumour‐derived chemokine that recruits protumour immune cells and accelerates tumour progression.^201^ In pancreatic cancer, lactate accumulation induces Lys63 lactylation of α‐endosulfine, activates the c‐Src/STAT3 signalling axis and enhances CCL2 secretion.^202^ Elevated CCL2 signalling is associated with increased expression of immunosuppressive mediators across multiple immune subsets, including CD25 and IL‐10 in Treg cells, PD‐L2 and IL‐10 in TAMs, and PD‐L1, ARG1 and iNOS in MDSCs.^203^, ^204^, ^205^ Notably, reactive nitrogen species produced by intra‐tumoural myeloid cells nitrate CCL2, preventing Teff cell infiltration into the tumour parenchyma and confining these cells to stromal regions, thereby establishing a spatial barrier of immune exclusion.^206^

Myeloid cells are a major source of IL‐6, IL‐1β and TNF‐α. Under conditions of chronic hypoxia and lactate accumulation, the acute antitumour functions of these cytokines diminish, whereas their sustained low‐level signalling is increasingly exploited by tumour cells to promote immune evasion.^207^ Mechanistically, IL‐6 activates the JAK/STAT3 signalling pathway to upregulate PFKFB3 and HK2, IL‐1β enhances Warburg metabolism through p38 signalling, and TNF‐α induces HK2 and GLUT1 through NF‐κB and HIF‐1α, respectively.^208^, ^209^, ^210^ Enhanced glycolysis, in turn, reduces tumour sensitivity to TNF‐α‐mediated cytotoxicity.^211^ Beyond metabolic reprogramming, these inflammatory cytokines also promote PD‐L1 expression.^212^, ^213^ The IL‐6/STAT3 signalling axis enhances non‐sense‐mediated mRNA decay through the associated protein SMG1, thereby impairing neoantigen presentation and reducing tumour immunogenicity.^214^ Concurrently, NF‐κB activation downstream of IL‐1β and TNF‐α drives antiapoptotic and stress‐adaptive programs, including BCL‐xL and cIAPs, conferring resistance to Teff cell‐ and NK cell‐mediated killing.^215^

TGF‐β secreted by Treg cells and TAMs also acts directly on tumour cells, and the metabolic advantages of these immune subsets further potentiate this signalling axis.^216^ Enhanced TGF‐β signalling reinforces glycolysis by upregulating PFKFB3, HK2 and GLUT1, induces PD‐L1 expression, and suppresses antigen‐processing machinery genes, including B2M, ERAP1 and TAP, thereby collectively promoting immune evasion.^217^, ^218^ Critically, TGF‐β functions as a central driver of EMT, mechanistically linking metabolic rewiring to enhanced metastatic potential and reduced responsiveness to ICIs.^219^

#### 3.3.3 Crosstalk among immune populations

IFN‐γ not only targets tumour cells but also directly regulates TAMs. Brief exposure to IFN‐γ rapidly enhances glycolysis in TAMs while suppressing OXPHOS. Glycolysis‐derived ATP is required to sustain the JAK/STAT1 signalling axis, thereby supporting iNOS induction and downstream proinflammatory responses.^220^ In addition, IFN‐γ promotes polarization towards an M1‐like phenotype and induces CXCL9 and CXCL10 production, thereby facilitating recruitment of Teff cells and NK cells.^221^, ^222^ However, impaired IFN‐γ signalling within the TME compromises these antitumour functions. Reduced IFN‐γ levels also diminish PD‐L1 expression, thereby lowering ICI efficacy.^223^

IL‐10, produced by multiple immune populations including Treg cells and TAMs, exerts broad suppressive effects on antitumour immunity.^224^ Elevated IL‐10 secretion has been linked to the metabolic adaptability of Treg cells within the TME.^225^ Within the TME, IL‐10 contributes to the establishment of a B lymphocyte‐induced maturation protein 1 (BLIMP1)‐dependent exhaustion program in intra‐tumoral CD8^+^ T cells by modulating expression of multiple inhibitory receptors and the exhaustion‐associated transcriptional signature.^226^ Consistent with this, IL‐10R blockade, particularly in combination with PD‐1 blockade, enhances CD8^+^ T cell effector function.^227^ Concurrently, IL‐10 suppresses TLR‐induced glycolytic reprogramming in DCs, impairing their maturation and reducing IL‐12 production.^228^ In NK cells, IL‐10 receptor‐dependent activation of STAT3 promotes autocrine IL‐10 feedback, thereby stabilizing an immunosuppressive phenotype.^229^ Notably, IL‐10 can partially restore exhausted T cell function by enhancing OXPHOS and metabolic fitness, underscoring the marked context dependence of IL‐10‐mediated metabolic regulation.^230^

TGF‐β secreted by Treg cells and TAMs also acts directly on CD8^+^ T cells. Activation of this pathway induces CD103 expression while repressing the Krüppel‐like factor 2 (KLF2)/sphingosine‐1‐phosphate receptor 1 (S1PR1) signalling axis, thereby promoting tissue residency and retention of CD8^+^ T cells within the tumour stroma or peripheral tumour regions and contributing to immune exclusion.^231^

**FIGURE 3:**
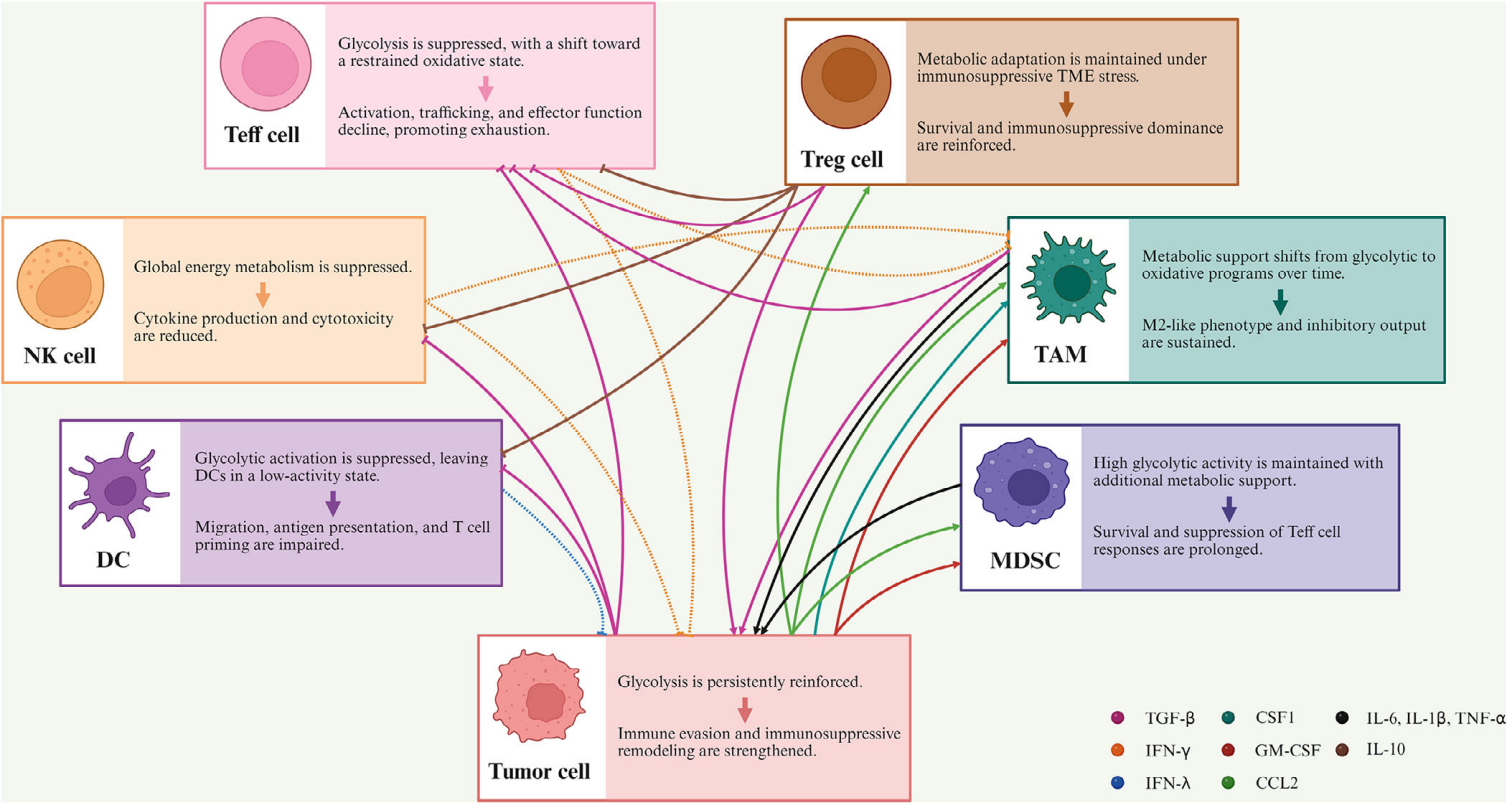
Immune consequences of reciprocal regulation between glucose metabolism and cytokine signalling. Glucose metabolic reprogramming shapes tumour immunity through cytokine‐linked signalling networks that suppress antitumour immune responses while sustaining immunosuppressive cell programs. Solid arrows indicate upregulated pathways or functions, whereas dashed arrows indicate downregulated pathways or functions.

Collectively, the interplay between metabolic pathways and cytokine signalling constitutes a systems‐level amplification module that confers directionality and durability on immune reprogramming within the TME (Figure 3). By forming self‐reinforcing regulatory circuits rather than acting as parallel influences, these two layers jointly determine whether tumour immunity is sustained, redirected, or extinguished.

### 3.4 Glycosylation

#### 3.4.1 Promotion of tumour cell immune evasion

In recent years, glycosylation has emerged as a critical regulator of tumour immunity. Beyond supplying energy, glucose metabolism also provides activated sugar donors required for glycan biosynthesis, thereby translating metabolic reprogramming into distinct glycosylation landscapes and further reshaping the immunometabolic network within the TME. Under conditions of aberrantly elevated glucose metabolism in tumour cells, multiple glycosylation pathways are concomitantly enhanced. Among these, the hexosamine biosynthetic pathway (HBP) serves as a key metabolic hub linking glucose metabolism to glycosylation. Through its rate‐limiting enzyme GFAT1, the HBP promotes uridine diphosphate N‐acetylglucosamine (UDP‐GlcNAc) generation, thereby providing substrate support for PD‐L1 N‐glycosylation and subsequent glycan processing.^232^ The glycosylation status of PD‐L1 is critical both for its stable membrane expression and for its functional interaction with PD‐1.^233^ This mechanism may partly explain why high guanosine monophosphate synthase (GMPS) expression in HCC suppresses the antitumour activity of CD8^+^ T cells and promotes immune evasion by enhancing PD‐L1 N‐glycosylation.^234^ More importantly, PD‐L1 N‐glycosylation is associated with reduced sensitivity to ICIs.^235^ Notably, the N35 site may have distinct regulatory significance because its loss can sustain PD‐1 signalling by promoting release of soluble PD‐L1, thereby paradoxically attenuating responsiveness to ICIs. PD‐L1 glycosylation status may therefore represent an important biomarker for predicting the efficacy of immunotherapy. Beyond PD‐L1, glycosylation may also contribute to the adenosinergic immunosuppressive axis. In TNBC, α‐1,3‐mannosyl‐glycoprotein 2‐β‐N‐acetylglucosaminyltransferase (MGAT1)‐mediated complex N‐glycosylation helps maintain stable membrane expression of CD73.^236^ Aberrant sialylation is even more prominent within the TME. Tumour cells and CAFs can remodel cell‐surface glycan structures by upregulating sialyltransferases, particularly members of the ST3 β‐galactoside α‐2,3‐sialyltransferase (ST3GAL) family.^237^, ^238^, ^239^ The resulting hypersialylated phenotype strengthens engagement with inhibitory Siglec receptors, suppresses Teff cell proliferation and activation, and promotes the transition of CD8^+^ T cells towards an exhausted‐like state. In PDAC, CAF‐derived sialylation signals can further drive differentiation of monocytes into immunosuppressive TAMs, thereby establishing a multicellular immunosuppressive network within the TME.^240^, ^241^ Accordingly, targeting interactions between sialylated glycans and glycoimmune checkpoint receptors has emerged as a potential new dimension of cancer immunotherapy.^242^ However, this strategy remains at the proof‐of‐concept stage, with evidence largely derived from early in vivo studies, and its clinical feasibility and safety require further evaluation.

#### 3.4.2 Constraints on antitumour immune populations

In Teff cells, stable membrane expression of inhibitory molecules, including PD‐1, CTLA‐4 and TIGIT, likewise depends on glycosylation.^243^, ^244^ Glycosylation‐mediated regulation of PD‐1 and PD‐L1 shares certain common features.^245^ Notably, the functional consequences of PD‐L1 glycosylation generally become evident only after global deglycosylation. By contrast, PD‐1 is more sensitive to site‐specific glycosylation, and even single‐site mutations produce marked effects. Moreover, the binding activities of camrelizumab and cemiplimab show clear dependence on PD‐1 fucosylation.^246^ From a therapeutic perspective, PD‐L1 N‐glycosylation may primarily affect antibody accessibility by increasing steric hindrance, whereas PD‐1 N‐glycosylation may more directly alter antibody recognition by reshaping antibody‐binding epitopes. Within the TME, competition for biosynthetic substrates preferentially constrains the glycosylation processes required to sustain Teff cell activation and functional adaptation, thereby weakening antitumour immunity. In this context, precise modulation of key glycosylation events is increasingly regarded as a potential strategy for reprogramming tumour immune responses. For example, short‐term fasting alleviates T cell exhaustion in HCC by suppressing CD36 N‐glycosylation.^247^ In addition, chronic inflammatory signals can remodel intra‐cellular glycosyltransferase networks. IL‐10 has been shown to upregulate MGAT5 through STAT3 activation, thereby enhancing N‐glycan branching on the surface of CD8^+^ T cells, increasing the threshold for TCR activation and reducing antigen sensitivity.^248^ Overall, the TME tends to selectively reconfigure the glycosylation landscape of Teff cells towards a hyporesponsive and functionally constrained state, ultimately exacerbating immune evasion.

#### 3.4.3 Reinforcement of protumour immune populations

Glycosylation is also an important mechanism for maintaining functional homeostasis in protumour immune cells. In Treg cells, high lactate concentrations can promote MGAT1 upregulation and mitochondrial translocation, which, in turn, mediate N‐glycosylation of progranulin and hypoxia‐upregulated 1 (HYOU1), stabilize mitochondrial membrane potential and metabolic homeostasis, and thereby strengthen immunosuppressive function.^249^ Future studies should further define the hierarchical organization of lactate‐associated post‐translational modification networks to determine whether Treg cell functional homeostasis is coregulated by multiple coordinated modifications. In TAMs, enhanced glucose metabolism can activate the HBP and increase O‐GlcNAcylation, thereby promoting stability, maturation and secretion of the lysosomal protease cathepsin B.^250^ Elevated cathepsin B activity helps maintain the immunosuppressive phenotype of TAMs and supports tumour immune evasion. This finding identifies a potential intervention point for improving current therapeutic responses, although its generalizability across tumour types remains to be established.

Overall, glycosylation represents a downstream molecular inscription mechanism through which glucose metabolic reprogramming is converted into persistent changes in receptor stability, signal integration and intercellular recognition. It therefore stabilizes the immunological consequences of metabolic remodelling by embedding metabolic information into the molecular architecture of cell‐to‐cell communication.

### 3.5 Reciprocal coupling between glucose metabolism and immune checkpoint signalling

#### 3.5.1 Metabolic regulation of immune checkpoint signalling

Glucose metabolism within the TME directly regulates immune checkpoint expression, primarily through the transcriptional and signalling activities of glycolysis‐associated enzymes. In breast cancer and glioblastoma, HK2 phosphorylates IκBα, promoting its rapid degradation and thereby activating NF‐κB signalling. Activated NF‐κB subsequently translocates to the nucleus and enhances PD‐L1 surface expression.^251^, ^252^ In PDAC, TAM‐derived TGF‐β1 induces conversion of PKM2 from the tetrameric to the dimeric conformation, facilitating its nuclear translocation. Nuclear PKM2 forms a complex with STAT1 that directly binds the PD‐L1 promoter, thereby enhancing PD‐L1 transcription.^253^ Consistently, in the CT26 syngeneic mouse model, pharmacological stabilization of PKM2 in its high‐activity tetrameric form with TEPP‐46, or silencing of PKM2 expression, markedly reduces PD‐L1 expression.^254^ Similarly, in oesophageal squamous cell carcinoma, PFKFB3 interacts with HIF‐1α, undergoes nuclear translocation and induces PD‐L1 expression, thereby facilitating immune evasion.^255^

Immune checkpoint regulation is further shaped by cellular energy status. Under metabolic stress, AMPK directly phosphorylates PD‐L1 at Ser195, inducing aberrant endoplasmic reticulum‐associated mannose trimming during PD‐L1 glycosylation and thereby reducing PD‐L1 expression.^256^ To date, most evidence for this mechanism has been derived from tumour cells, and whether analogous energy‐sensing pathways regulate immune checkpoint stability in immune cells remains an important question for future investigation.

#### 3.5.2 Metabolic effects of immune checkpoint signalling

Conversely, immune checkpoints themselves exert profound effects on cellular metabolism. PD‐1 and CTLA‐4 signalling suppress glycolytic metabolism in Teff cells, with PD‐1 additionally promoting FAO and lipolysis.^257^ Across multiple cancer types, activation of the CD155/TIGIT signalling axis markedly suppresses glucose metabolism in CD8^+^ T cells, and comparable metabolic inhibition has also been observed in NK cells.^258^, ^259^, ^260^ Further integrative analyses of immune checkpoint signalling and immune cell metabolic regulation will therefore be essential for identifying novel therapeutic vulnerabilities and optimizing checkpoint‐based immunotherapies.

Taken together, bidirectional crosstalk between cellular metabolism and immune checkpoint pathways forms a self‐reinforcing regulatory mechanism that couples bioenergetic state to inhibitory signalling. This reciprocity not only links metabolic stress to immune restraint but also consolidates chronic immune dysfunction, thereby entrenching resistance to productive antitumour immunity.

## 4 CONCLUSION AND FUTURE PERSPECTIVE

Glucose metabolic reprogramming within the TME should be understood not as a passive consequence of tumour growth but as a hierarchical immunoregulatory architecture that reshapes the local immune landscape by redistributing metabolic fitness between tumour cells and immune cell populations. Within this framework, glucose competition serves as an upstream gating mechanism that establishes a selective bioenergetic hierarchy. Lactate accumulation and reciprocal regulation between glucose metabolism and cytokine signalling further amplify immunosuppressive signalling and reinforce immune exclusion (Figures 2 and 3). Glycosylation converts altered metabolic flux into durable changes in receptor stability, ligand recognition and immune checkpoint responsiveness. Bidirectional coupling between cellular metabolism and immune checkpoint pathways subsequently consolidates these processes into chronic immune dysfunction and therapeutic resistance. Collectively, these mechanisms position tumour glucose metabolism as a central organizer of immune equilibrium, immune escape and therapeutic failure within the TME. Elucidating the mechanistic basis of this immunometabolic regulation provides a conceptual framework for the rational integration of metabolic interventions with immunotherapy, with the potential to enhance therapeutic precision, overcome resistance and achieve more durable clinical benefit.

Further development of metabolic intervention strategies must be grounded in a spatiotemporally precise framework. From a temporal perspective, mistimed intervention may not only impair the establishment of early antitumour immune responses and induce tolerogenic reprogramming but also deplete the memory precursor and stem‐like cell pools required for durable therapeutic benefit.^261^ In this context, the timing of metabolic intervention should, whenever possible, be defined dynamically through longitudinal biopsy‐based multiomics assessment integrated with serial metabolic imaging, rather than determined empirically according to fixed treatment schedules.^262^, ^263^ From a spatial perspective, insufficient resolution may cause single‐site sampling to underestimate or overlook dominant immunosuppressive niches. More importantly, metabolically targeted interventions that lack selectivity for lesions, microenvironmental compartments and specific cell populations may increase off‐tumour exposure and safety liabilities, thereby narrowing the effective therapeutic window.^264^, ^265^ Therefore, platforms that integrate spatial metabolomics with immune phenotyping at single‐cell resolution, together with metabolic imaging technologies that enable in vivo localization and delineation, should serve as important enabling tools for the design of combination regimens.^266^, ^267^ Nevertheless, substantial obstacles continue to impede the clinical translation of these advances.^268^, ^269^ Ultimately, the decisive factor determining whether metabolism‐targeted combination immunotherapy can enter routine clinical practice will not be the continued expansion of the catalogue of metabolic targets but the establishment of a spatiotemporal stratification framework capable of jointly guiding patient selection, intervention timing and lesion‐ or region‐level deployment.

## AUTHOR CONTRIBUTIONS

Conceptualized and designed the study, reviewed and edited the manuscript, acquired the funding and supervised the research: Jian Zhou and Xinrong Yang. Conducted the investigation, performed the visualization and prepared the original draft of the manuscript: Yang Wang and Yijun Lu.

## CONFLICT OF INTEREST STATEMENT

The authors declare no conflict of interest.

## Data Availability

Data sharing does not apply to this article as no datasets were generated or analyzed during the current study.
